# A Rapid Convergent Low Complexity Interference Alignment Algorithm for Wireless Sensor Networks

**DOI:** 10.3390/s150818526

**Published:** 2015-07-29

**Authors:** Lihui Jiang, Zhilu Wu, Guanghui Ren, Gangyi Wang, Nan Zhao

**Affiliations:** 1 School of Electronics and Information Engineering, Harbin Institute of Technology, Harbin 150001, China; E-Mails: jianglihui@hit.edu.cn (L.J.); rgh@hit.edu.cn (G.R.); 2 School of Instrumentation Science and Opto-electronics Engineering, Beihang University, Beijing 100191, China; E-Mail: WangGangyi@buaa.edu.cn; 3 School of Information and Communication Engineering, Dalian University of Technology, Dalian 116024, China; E-Mail: zhaonan@dlut.edu.cn

**Keywords:** wireless sensor networks, multiple-input and multiple-output (MIMO), interference channel, interference alignment, line search, iterative algorithms

## Abstract

Interference alignment (IA) is a novel technique that can effectively eliminate the interference and approach the sum capacity of wireless sensor networks (WSNs) when the signal-to-noise ratio (SNR) is high, by casting the desired signal and interference into different signal subspaces. The traditional alternating minimization interference leakage (AMIL) algorithm for IA shows good performance in high SNR regimes, however, the complexity of the AMIL algorithm increases dramatically as the number of users and antennas increases, posing limits to its applications in the practical systems. In this paper, a novel IA algorithm, called directional quartic optimal (DQO) algorithm, is proposed to minimize the interference leakage with rapid convergence and low complexity. The properties of the AMIL algorithm are investigated, and it is discovered that the difference between the two consecutive iteration results of the AMIL algorithm will approximately point to the convergence solution when the precoding and decoding matrices obtained from the intermediate iterations are sufficiently close to their convergence values. Based on this important property, the proposed DQO algorithm employs the line search procedure so that it can converge to the destination directly. In addition, the optimal step size can be determined analytically by optimizing a quartic function. Numerical results show that the proposed DQO algorithm can suppress the interference leakage more rapidly than the traditional AMIL algorithm, and can achieve the same level of sum rate as that of AMIL algorithm with far less iterations and execution time.

## 1. Introduction

Wireless sensor networks (WSNs) have received considerable attention, and have been applied to a large number of scenarios in recent years [1]. With the growth in network scale, interference management has become one of the major challenges in WSNs where multiple users often share some common resources simultaneously and high data rates are often demanded. A large amount of research has focused on interference management techniques in order to increase the sum rate of the system [2,3,4,5]. One recent interesting interference management scheme that can approach the channel capacity is interference alignment (IA), which was firstly studied by Maddah-Ali *et al.* [6] as well as Jafar and Shamai [7] for the multiple-input and multiple-output (MIMO) X channel. Subsequently, Cadambe and Jafar [8] proved that the throughput of the system varies linearly with the number of users in high signal-to-noise ratio (SNR) regimes by employing IA. In addition, the degrees of freedom (DoFs), which serve as one key measure of the channel capacity, were studied by Lee *et al.* [9] in the case of the MIMO Y channel and by Cadambe and Jafar [10] in the case of relay networks. Thanks to its bright prospects IA has been extended to use in many scenarios such as OFDM systems [11], femtocell networks [12], and cognitive networks [13,14]. In particular, Wu *et al.* [15] have leveraged the IA technique to mitigate the strong interference in large scale WSNs. In heterogeneous networks, Sharma *et al.* [16] have proposed a novel spectral coexistence mechanism which takes advantage of the IA technique.

Essentially, achieving IA is to design proper transmitting precoding matrices **V** to align the desired signal and interference to different signal subspaces, and to design proper receiving decoding matrices **U** to eliminate the interference at each receiver [17]. Tresch *et al.* [18] have developed closed-form solutions to IA for the MIMO interference channel with *M* + 1 users, one degree of freedom, *M* transmitting antennas, and *M* receiving antennas. However, the closed-form solutions to IA remain unknown except for some special situations [19]. Peters and Heath [20] have shown that it is difficult to formulate the closed-form solutions of **V** and **U** in the situation of more than three users. In addition, Razaviyayn *et al.* [21] have also shown that searching the optimal **V** and **U** is a nonconvex problem. Ma *et al.* [22] have performed a deep analysis on the computational complexity of interference alignment, and shown that the problem becomes NP-hard when the number of antennas at each node is larger than two. Therefore, many researchers [20,21,22,23,24,25,26] have focused on numerical methods to find the suboptimal **V** and **U**. Gomadam *et al.* [23] have leveraged the channel reciprocity and proposed the alternating minimization interference leakage (AMIL) and Max-SINR algorithms, which only require local channel state information (CSI). It has been shown by Xu *et al.* [27] that the AMIL and Max-SINR algorithms show close multiplexing gains in high SNR regimes and the former approach has lower complexity than the latter one. A similar alternating minimization IA algorithm has been proposed in [20] without assuming the network reciprocity. Shen *et al.* [24] have developed the minimum mean square error (MMSE) IA algorithm for the case of imperfect CSI. A sequential antenna switch algorithm was proposed in [28], in which the quality of service of the IA system was considered.

Among these methods, the AMIL algorithm can suppress the interference leakage to a low level and thus can achieve high throughput in high SNR regimes where the sum rate of the system is determined by interference instead of noise. However, it might take a large number of iterations as well as long computational time in the case of large numbers of users and antennas. Furthermore, for the reason that CSI is time-variable, the practical IA system has limited computational time, which will certainly become the bottleneck of the implementation of AMIL algorithm when there are plenty of users and antennas. In this paper, we investigate the properties of the AMIL algorithm, and discover that the difference between the consecutive iteration results of the AMIL algorithm will approximately point to the convergence solution when the precoding and decoding matrices obtained from the intermediate iterations are sufficiently close to their convergence values. Based on this property, we propose a rapidly convergent low-complexity IA algorithm, *i.e.*, directional quartic optimal (DQO) algorithm. It leverages a line search (LS) optimization method, which iteratively generates searching directions and optimal step sizes. The searching direction is obtained by subtracting the consecutive results of AMIL algorithm, and the optimal step size is calculated by solving a quartic optimization problem.

The rest of the paper is organized as follows: in Section 2, we describe the system model. The properties of the AMIL algorithm are studied in Section 3, which will serve as the foundation of the algorithm proposed later. In Section 4, the DQO algorithm is proposed, and the corresponding procedure, optimal step size calculation, and complexity analysis are provided. Numerical results to evaluate the proposed algorithm are presented and discussed in Section 5. Finally, the paper is concluded in Section 6. As far as the notation used in the paper is concerned, we employ (*M* × *N*, *d*)*^K^* to represent a *K*-user MIMO interference channel where each user wishes to transmit *d* data streams with *M* transmitting antennas and *N* receiving antennas. We use C, R, C*^M^*^×*N*^, **I**, and CN(μ, σ^2^) to represent the complex domain, the real domain, the *M* × *N* complex matrix, the identity matrix, and the complex Gaussian distribution with mean μ and variance σ^2^, respectively. Re{*a*} denotes the real part of scalar *a*. **A**^T^, A*, **A**^H^, ||**A**||, Tr[**A**] and A_*(*l*)_ mean the transpose, the conjugate, the conjugate transpose, the Frobenius norm, the trace and the *l*-th column of matrix **A**, respectively. eig*_l_*[**A**] stands for the eigenvector associated with the *l*-th smallest eigenvalue of matrix **A**. We employ **A***^i^* to represent the value of **A** at the *i*-th iterations. *diag*(*a*_1_, *a*_2_, ..., *a*_m_) represents a diagonal matrix with its diagonal elements equal to *a*_1_, *a*_2_, …, *a*_m_.

## 2. System Model

**Figure 1:**
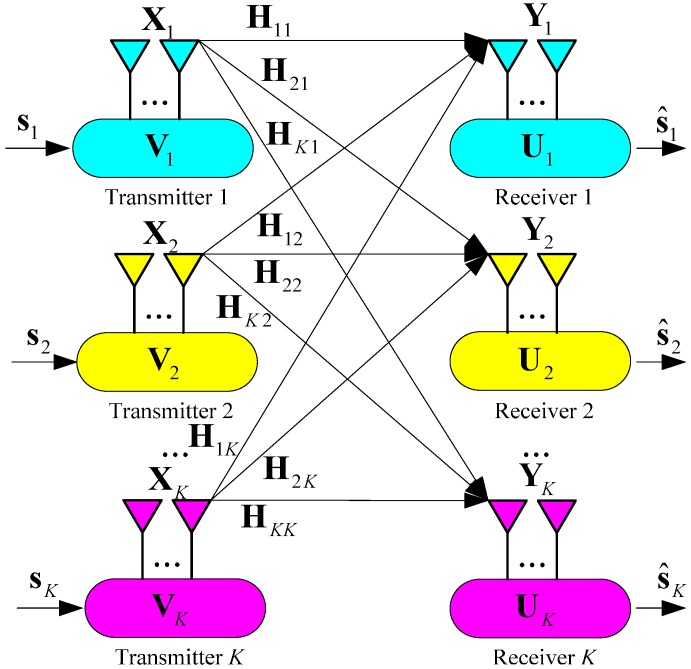
System model of the *K*-user MIMO interference channel.

In this paper, the (*M* × *N*, *d*)*^K^* MIMO interference channel is considered, and is depicted in Figure 1. The received signal of the *k*-th user can be represented as [23]:

(1) $$Y_{k}=\sum_{l=1}^{K}H_{kl}V_{l}s_{l}+n_{k},k\in 1,2,...,K$$

where **s***_k_* ∈ C*^d^*^×1^, **V***_k_* ∈ C*^M^*^×*d*^, and **Y***_k_* ∈ C*^N^*^×1^ denote the data vector, the precoding matrix, and the received signal vector of the *k*-th user, respectively; **n***_k_* ∈ C*^N^*^×1^ represents the noise vector with distribution of CN(0, σ^2^) for each element; **H***_kl_* ∈ C*^N^*^×*M*^ represents the channel matrix from transmitter *l* to receiver *k*. In the network we considered, CSI is obtained through CSI feedback, which serves as one critical technique in IA. A large number of researchers such as Ayach [29], Cho [30], and Zhang [31] have focused on the CSI feedback strategy. Since we mainly focus on designing the precoding and decoding matrices, further investigation on CSI feedback is beyond the scope of the paper and an accurate global CSI is assumed to be available at each node throughout this paper.

The *k*-th user’s covariance matrices of the forward and reciprocal networks are respectively given by:

(2) $$Q_{k}=\sum_{l=1,l\neq k}^{K}\frac{P_{l}}{d}H_{kl}V_{l}V_{l}^{\text{H}}H_{kl}^{\text{H}}$$

(3) $${\overset{\leftarrow }{Q}}_{k}=\sum_{l=1,l\neq k}^{K}\frac{{\overset{\leftarrow }{P}}_{l}}{d}H_{lk}^{\text{H}}U_{l}U_{l}^{\text{H}}H_{lk}$$

where *P_l_* and $\overset{\leftarrow }{P}$ denote the transmitting power of the *l*-th user in the forward and the reciprocal networks, respectively. Then the interference leakage at receiver *k* can be calculated as:

(4) $$L_{k}=\text{Tr}[U_{k}^{\text{H}}Q_{k}U_{k}]$$

where **U***_k_* ∈ C*^N^*^×*d*^ is the decoding matrix at receiver *k*. The total interference leakage is defined as the sum of the leakage at each receiver, *i.e.*,:

(5) $$L=\sum_{k=1}^{K}L_{k}$$

Then the minimal interference leakage problem is to design proper transmitting precoding matrices **V** and receiving decoding matrices **U** to minimize Equation (5) under the following constraints:

(6) $$\text{rank}(U_{k}^{\text{H}}H_{kk}V_{k})=d$$

(7) $$V_{k}^{\text{H}}V_{k}=I,U_{k}^{\text{H}}U_{k}=I$$

The former constraint guarantees the feasibility of the desired signal reconstruction and the later one ensures the uniqueness of the IA solution [17].

## 3. Properties of AMIL Algorithm

The AMIL algorithm, whose procedure is summarized in Algorithm 1, was proposed by Gomadam *et al.* [23] to find the IA solution by exploiting the channel reciprocity. In this section, we investigate the properties of the AMIL algorithm, and show that the difference of the consecutive iteration results of AMIL algorithm will approximately point to the convergence solution when the precoding and decoding matrices obtained from the intermediate iterations are sufficiently close to their convergence values. This interesting property will serve as the foundation of the algorithm proposed later.

**Algorithm 1** AMIL Algorithm
Initialize *L*_th_, *I*_max_, and $V_{k}^{0}$, with ${\left(V_{k}^{0}\right)}^{\text{H}}V_{k}^{0}=I$ (*k* = 1, 2,…, *K*).*i *= 0.**Repeat***i* = *i* + 1(VU-step):Calculate $Q_{k}^{i}$ according to Equation (2). Update $U_{k}^{i}$ so that$U_{k(l)}^{i}={\text{eig}}_{l}[Q_{k}^{i}],l=1,2,..,d.$(UV-step):Calculate $\overset{\leftarrow }{Q_{k}^{i}}$ according to Equation (3). Update $V_{k}^{i}$ so that$V_{k(l)}^{i}={\text{eig}}_{l}[\overset{\leftarrow }{Q_{k}^{i}}],l=1,2,..,d.$Calculate the interference leakage *L* according to Equation (5).**Until** *L* < *L*_th_ or *i* > *I*_max_.


As shown in Algorithm 1, the AMIL algorithm calculates the optimal decoding matrices **U** by fixing **V** in the forward channel, and computes the optimal **V** by fixing **U** in the reciprocal channel. It can monotonously suppress the interference leakage, and has been proved to converge to a local minimum. Although the convergence point is not guaranteed to be the optimal IA solution, it has been verified numerically that in many cases AMIL algorithm can achieve a low interference leakage level after sufficient iterations. Therefore, in high SNR regimes, where the sum rate is limited by interference rather than noise, the AMIL algorithm serves as a good method to increase the sum rate. In addition, the procedure of alternating minimization is very effective, and is often employed by many other algorithms. In spite of this, the iterations as well as time required by AMIL algorithm to reach a certain level of interference leakage increase dramatically with the number of users and antennas. Therefore, the properties of AMIL algorithm are studied in order to figure out a way to increase the convergence rate and thus to reduce its complexity as well as the required computational time.

Throughout this paper, $V_{k}^{i}$ ∈ C^*M*×*d*^ and $U_{k}^{i}$ ∈ C^*N*×*d*^ are employed to represent the *k*-th user’s precoding and decoding matrices obtained from the *i*-th iteration, respectively. The convergence value of $V_{k}^{i}$ and $U_{k}^{i}$ are defined as ${\tilde{V}}_{k}$ and ${\tilde{U}}_{k}$, respectively. Define $\Delta V_{k}^{i}$ ∈ C^*M*×*d*^ as the deviation from $V_{k}^{i}$ to ${\tilde{V}}_{k}$, and $\Delta U_{k}^{i}$ ∈ C^*N*×*d*^ as the deviation from $U_{k}^{i}$ to ${\tilde{U}}_{k}$ so that:

(8) $$V_{k}^{i}={\tilde{V}}_{k}+\Delta V_{k}^{i}$$

(9) $$U_{k}^{i}={\tilde{U}}_{k}+\Delta U_{k}^{i}$$

Define ${\bar{V}}^{i}$, $\bar{\tilde{V}}$, $\Delta {\bar{V}}^{i}$∈ C^*MdK*×1^ and ${\bar{U}}^{i}$, $\bar{\tilde{U}}$, $\Delta {\bar{U}}^{i}$∈ C*^NdK^*^×^^1^ as:

(10) $${\bar{V}}^{i}=\left[\begin{array}{l} V_{1(1)}^{i}\\ V_{1(2)}^{i}\\ ...\\ V_{1(d)}^{i}\\ ...\\ V_{K(1)}^{i}\\ V_{K(2)}^{i}\\ ...\\ V_{K(d)}^{i} \end{array}\right],\bar{\tilde{V}}=\left[\begin{array}{l} {\tilde{V}}_{1(1)}\\ {\tilde{V}}_{1(2)}\\ ...\\ {\tilde{V}}_{1(d)}\\ ...\\ {\tilde{V}}_{K(1)}\\ {\tilde{V}}_{K(2)}\\ ...\\ {\tilde{V}}_{K(d)} \end{array}\right],\Delta {\bar{V}}^{i}=\left[\begin{array}{l} \Delta V_{1(1)}^{i}\\ \Delta V_{1(2)}^{i}\\ ...\\ \Delta V_{1(d)}^{i}\\ ...\\ \Delta V_{K(1)}^{i}\\ \Delta V_{K(2)}^{i}\\ ...\\ \Delta V_{K(d)}^{i} \end{array}\right],{\bar{U}}^{i}=\left[\begin{array}{l} U_{1(1)}^{i}\\ U_{1(2)}^{i}\\ ...\\ U_{1(d)}^{i}\\ ...\\ U_{K(1)}^{i}\\ U_{K(2)}^{i}\\ ...\\ U_{K(d)}^{i} \end{array}\right],\bar{\tilde{U}}=\left[\begin{array}{l} {\tilde{U}}_{1(1)}\\ {\tilde{U}}_{1(2)}\\ ...\\ {\tilde{U}}_{1(d)}\\ ...\\ {\tilde{U}}_{K(1)}\\ {\tilde{U}}_{K(2)}\\ ...\\ {\tilde{U}}_{K(d)} \end{array}\right],\Delta {\bar{U}}^{i}=\left[\begin{array}{l} \Delta U_{1(1)}^{i}\\ \Delta U_{1(2)}^{i}\\ ...\\ \Delta U_{1(d)}^{i}\\ ...\\ \Delta U_{K(1)}^{i}\\ \Delta U_{K(2)}^{i}\\ ...\\ \Delta U_{K(d)}^{i} \end{array}\right]$$

From Equations (8) and (9), it can be obtained that:

(11) $${\bar{V}}^{i}=\bar{\tilde{V}}+\Delta {\bar{V}}^{i}$$

(12) $${\bar{U}}^{i}=\bar{\tilde{U}}+\Delta {\bar{U}}^{i}$$

The investigation begins with the linear transformation property of AMIL algorithm as follows:

**Theorem** **1.** *If AMIL algorithm can converge to a point where the interference leakage is very small, there exist fixed linear transformations **T**_V_ and **T**_U_, which do not change with iterations. When ${\bar{V}}^{i}$* and *${\bar{U}}^{i}$* are sufficiently close to *$\bar{\tilde{V}}$* and *$\bar{\tilde{U}}$, respectively, the deviations of the (i + 1)-th iteration can be approximated as follows*:

(13) $$\Delta {\bar{V}}^{i+1}\approx T_{\text{V}}\Delta {\bar{V}}^{i}$$

(14) $$\Delta {\bar{U}}^{i+1}\approx T_{\text{U}}\Delta {\bar{U}}^{i}$$

**Proof** **of Theorem 1.** In the situation when AMIL algorithm converges to a point where the interference leakage is very small, which is easy to achieve for AMIL algorithm, we have:

(15) $${\tilde{U}}_{j}^{\text{H}}H_{ji}{\tilde{V}}_{i}\approx 0,j\neq i$$

Define the interference covariance matrices at the convergence point as:

(16) $${\tilde{Q}}_{k}=\sum_{j=1,j\neq k}^{K}H_{kj}{\tilde{V}}_{j}{\tilde{V}}_{j}^{\text{H}}H_{kj}^{\text{H}}$$

(17) $${\overset{\leftarrow }{\tilde{Q}}}_{k}=\sum_{j=1,j\neq k}^{K}H_{jk}^{\text{H}}{\tilde{U}}_{j}{\tilde{U}}_{j}^{\text{H}}H_{jk}$$

Assume that the *l*-th smallest eigenvalue and the associated eigenvector of ${\tilde{Q}}_{k}$ are $\overset{\leftarrow }{\lambda_{k,l}}$ and ${\overset{\leftarrow }{\nu }}_{k,l}$, respectively. Similarly for ${\overset{\leftarrow }{\tilde{Q}}}_{k}$, we have $\lambda_{k,l}$ and $\nu_{k,l}$. From the procedure of AMIL algorithm, ${\tilde{V}}_{k}$ and ${\tilde{U}}_{k}$ should be the eigenvectors corresponding to the *d* smallest eigenvalues of ${\overset{\leftarrow }{\tilde{Q}}}_{k}$ and ${\tilde{Q}}_{k}$, respectively. Therefore, ${\tilde{V}}_{k}$ and ${\tilde{U}}_{k}$ can be respectively given by:

(18) $${\tilde{V}}_{k}=[\nu_{k,1},\nu_{k,2},...,\nu_{k,d}]$$

(19) $${\tilde{U}}_{k}=[{\overset{\leftarrow }{\nu }}_{k,1},{\overset{\leftarrow }{\nu }}_{k,2},...,{\overset{\leftarrow }{\nu }}_{k,d}]$$

At the (*i* + 1)-th iteration, the VU-step of AMIL algorithm is performed on the precoding matrices $\left\{V_{k}^{i}\right\}$ and covariance matrix at the (*i* + 1)-th iteration can be expressed as:

(20) $$\begin{array}{cc} Q_{k}^{i+1} & =\sum_{j=1,j\neq k}^{K}H_{kj}V_{j}^{i}V_{j}^{i\text{H}}H_{kj}^{\text{H}}\\ & =\sum_{j=1,j\neq k}^{K}H_{kj}[{\tilde{V}}_{j}+\Delta V_{j}^{i}]{\left[{\tilde{V}}_{j}+\Delta V_{j}^{i}\right]}^{\text{H}}H_{kj}^{\text{H}}\\ & \text{=}{\tilde{Q}}_{k}\text{+}\Delta Q_{k}^{i+1} \end{array}$$

where $\Delta Q_{k}^{i+1}$ is given by:

(21) $$\Delta Q_{k}^{i+1}\;=\sum_{j=1,j\neq k}^{K}H_{kj}[\Delta V_{j}^{i}{\tilde{V}}_{j}^{\text{H}}+{\tilde{V}}_{j}\Delta V_{j}^{i\text{H}}+\Delta V_{j}^{i}\Delta V_{j}^{i\text{H}}]H_{kj}^{\text{H}}$$

When ${\bar{V}}^{i}$ and ${\bar{U}}^{i}$ are sufficiently close to $\bar{\tilde{V}}$ and $\bar{\tilde{U}}$ respectively, the deviation $\Delta V_{j}^{i}$ is very small. Therefore, the high order term $\Delta V_{j}^{i}\Delta V_{j}^{i\text{H}}$ can be ignored, and Equation (21) can be simplified as:

(22) $$\Delta Q_{k}^{i+1}\;\approx \sum_{j=1,j\neq k}^{K}H_{kj}[\Delta V_{j}^{i}{\tilde{V}}_{j}^{\text{H}}+{\tilde{V}}_{j}\Delta V_{j}^{i\text{H}}]H_{kj}^{\text{H}}$$

According to the AMIL algorithm, ${\text{U}}_{k}^{i+1}$ should be the eigenvector corresponding to the *d* smallest eigenvalues of $Q_{k}^{i+1}$. From Equations (19) and (20), using the Eigenvalue Perturbation Theory, the *l*-th column of $U_{k}^{i+1}$ can be approximated by:

(23) $$\begin{array}{cc} U_{k(l)}^{i+1} & \approx {\overset{\leftarrow }{\nu }}_{k,l}+\sum_{j=1,j\neq l}^{N}\frac{{\overset{\leftarrow }{\nu }}_{k,j}^{\text{H}}\Delta Q_{k}^{i+1}{\overset{\leftarrow }{\nu }}_{k,l}}{\overset{\leftarrow }{\lambda_{k,l}}-\overset{\leftarrow }{\lambda_{k,j}}}{\overset{\leftarrow }{\nu }}_{k,j}\\ & ={\tilde{U}}_{k(l)}+\sum_{j=1,j\neq l}^{N}\frac{{\overset{\leftarrow }{\nu }}_{k,j}^{\text{H}}\Delta Q_{k}^{i+1}{\tilde{U}}_{k(l)}}{\overset{\leftarrow }{\lambda_{k,l}}-\overset{\leftarrow }{\lambda_{k,j}}}{\overset{\leftarrow }{\nu }}_{k,j}\\ & ={\tilde{U}}_{k(l)}+\Delta U_{k(l)}^{i+1} \end{array}$$

where $\Delta U_{k(l)}^{i+1}$ is given by:

(24) $$\Delta U_{k(l)}^{i+1}=\sum_{j=1,j\neq l}^{N}\frac{{\overset{\leftarrow }{\nu }}_{k,j}^{\text{H}}\Delta Q_{k}^{i+1}{\tilde{U}}_{k(l)}}{\overset{\leftarrow }{\lambda_{k,l}}-\overset{\leftarrow }{\lambda_{k,j}}}{\overset{\leftarrow }{\nu }}_{k,j}$$

Substitute Equation (22) into (24), and $\Delta U_{k(l)}^{i+1}$ can be rewritten as:

(25) $$\Delta U_{k(l)}^{i+1}\;\approx \sum_{j=1,j\neq l}^{N}\frac{1}{\overset{\leftarrow }{\lambda_{k,l}}-\overset{\leftarrow }{\lambda_{k,j}}}{\overset{\leftarrow }{\nu }}_{k,j}^{\text{H}}\{\sum_{r=1,r\neq k}^{K}H_{kr}\;[\Delta V_{r}^{i}{\tilde{V}}_{r}^{\text{H}}+{\tilde{V}}_{r}\Delta V_{r}^{i\text{H}}]H_{kr}^{\text{H}}\}{\tilde{U}}_{k(l)}{\overset{\leftarrow }{\nu }}_{k,j}$$

From Equation (15), the terms ${\tilde{V}}_{r}^{\text{H}}H_{kr}^{\text{H}}{\tilde{U}}_{k(l)}$ in Equation (25) is approximately zero and thus can be omitted. Therefore, Equation (25) can be simplified as:

(26) $$\Delta U_{k(l)}^{i+1}\approx \sum_{j=1,j\neq l}^{N}\frac{{\overset{\leftarrow }{\nu }}_{k,j}^{\text{H}}\sum_{r=1,r\neq k}^{K}H_{kr}{\tilde{V}}_{r}\Delta V_{r}^{i\text{H}}H_{kr}^{\text{H}}{\tilde{U}}_{k(l)}}{\overset{\leftarrow }{\lambda_{k,l}}-\overset{\leftarrow }{\lambda_{k,j}}}{\overset{\leftarrow }{\nu }}_{k,j}$$

From Equation (26), it can be seen that $\Delta U_{k(l)}^{i+1}$ is approximately the linear combination of the elements of ${\left(\Delta V_{r}^{i}\right)}^{*}$, and the coefficients of the combination are corresponding to ${\overset{\leftarrow }{\nu }}_{k,j}^{\text{H}}$, $H_{kr}$, ${\tilde{V}}_{r}$, ${\tilde{U}}_{k}$ and $\overset{\leftarrow }{\lambda_{k,j}}$, which are fixed and do not vary with iterations *i*. Hence there exists a fixed matrix **T**_VU_ ∈ C*^NKd^* ^×^^*MKd*^ which does not change with iterations, so that:

(27) $$\Delta {\bar{U}}^{i+1}\approx T_{\text{VU}}\cdot {\left(\Delta {\bar{V}}^{i}\right)}^{*}$$

Similarly for the UV-step, there exists a fixed matrix **T**_UV_ ∈ C*^MKd^* ^× *NKd*^ which does not change with iterations, so that:

(28) $$\Delta {\bar{V}}^{i+1}\approx T_{\text{UV}}\cdot {\left(\Delta {\bar{U}}^{i+1}\right)}^{*}$$

From Equations (27) and (28), we have:

(29) $$\Delta {\bar{V}}^{i+1}\approx T_{\text{UV}}T_{\text{VU}}^{*}\cdot \Delta {\bar{V}}^{i}=T_{\text{V}}\Delta {\bar{V}}^{i}$$

(30) $$\Delta {\bar{U}}^{i+1}\approx T_{\text{VU}}T_{\text{UV}}^{*}\cdot \Delta {\bar{U}}^{i}=T_{\text{U}}\Delta {\bar{U}}^{i}$$

where:

(31) $$T_{\text{V}}=T_{\text{UV}}T_{\text{VU}}^{*}\in {\mathbb{C}}^{MKd\times MKd}$$

(32) $$T_{\text{U}}=T_{\text{VU}}T_{\text{UV}}^{*}\in {\mathbb{C}}^{NKd\times NKd}$$

It can be seen that **T**_V_ and **T**_U_ determine the properties of AMIL algorithm when the current point is sufficiently close to its convergence value. As the analytical forms of **T**_V_ and **T**_U_ are extremely complex, the features of these two transformations are investigated by simulation and one interesting property observed is given as follows:

**Property** **1.** *In the case of (M × M, d)^K^ channel, there exist two nonsingular matrices **P**_V_ and **P**_U_, so that **T**_V_ and **T**_U_ can be expressed as*:

(33) $$T_{\text{V}}=P_{\text{V}}^{-1}\Lambda P_{\text{V}}$$

(34) $$T_{\text{U}}=P_{\text{U}}^{-1}\Lambda P_{\text{U}}$$

*where **Λ** = diag(κ_1_, κ_2_,…, κ_MKd_) with κ_1_ > κ_2_ ... > κ_r_ and κ_r + 1_ = κ_r + 2_ = ... = κ_MKd_ = 0 (1 < r < KMd).*

Based on the properties above, we further study the situation when consecutive applications of **T**_V_ and **T**_U_ are exerted on the deviation, and come to the following theorem.

**Theorem** **2.** *In the (M × M, d)^K^ channel, if AMIL algorithm can converge to a point where the interference leakage is very small, the convergence solutions ${\tilde{V}}_{k}$ and ${\tilde{U}}_{k}$ (k = 1, 2, ..., K) can be approximated by the following Equations (35) and (36), when $V_{k}^{i}$ and $U_{k}^{i}$ are sufficiently close to ${\tilde{V}}_{k}$ and ${\tilde{U}}_{k}$, respectively *:

(35) $${\tilde{V}}_{k}\approx V_{k}^{i}+t(V_{k}^{i}-V_{k}^{i-1}),t\in \mathbb{R}$$

(36) $${\tilde{U}}_{k}\approx U_{k}^{i}+t(U_{k}^{i}-U_{k}^{i-1})$$

**Proof** **of Theorem 2.** At the (*i* + *i*_1_)-th iteration, from Equations (29), (30), (33) and (34), we have:

(37) $$\begin{array}{cc} \Delta {\bar{V}}^{i+i_{1}} & \approx T_{\text{V}}^{i_{1}}\Delta {\bar{V}}^{i}\\ & =P_{\text{V}}^{-1}\Lambda^{i_{1}}P_{\text{V}}\Delta {\bar{V}}^{i}\\ & =P_{\text{V}}^{-1}diag(\kappa_{1}^{i_{1}},\kappa_{2}^{i_{1}},...,\kappa_{MKd}^{i_{1}})P_{\text{V}}\Delta {\bar{V}}^{i} \end{array}$$

(38) $$\begin{array}{cc} \Delta {\bar{U}}^{i+i_{1}} & \approx T_{\text{U}}^{i_{1}}\Delta {\bar{U}}^{i}\\ & =P_{\text{U}}^{-1}\Lambda^{i_{1}}P_{\text{U}}\Delta {\bar{U}}^{i}\\ & =P_{\text{U}}^{-1}diag(\kappa_{1}^{i_{1}},\kappa_{2}^{i_{1}},...,\kappa_{MKd}^{i_{1}})P_{\text{U}}\Delta {\bar{U}}^{i} \end{array}$$

Define **e***_k_*, **g***_k_* ∈ C*^MKd^*^×1^ and *f_k_*, *s_k_* ∈ C so that:

(39) $$P_{\text{V}}^{-1}=[e_{1},e_{2},...,e_{MKd}]$$

(40) $$P_{\text{U}}^{-1}=[g_{1},g_{2},...,g_{MKd}]$$

(41) $$P_{\text{V}}\Delta {\bar{V}}^{i}={\left[f_{1},f_{2},...,f_{MKd}\right]}^{\text{T}}$$

(42) $$P_{\text{U}}\Delta {\bar{U}}^{i}={\left[h_{1},h_{2},...,h_{MKd}\right]}^{\text{T}}$$

Thus Equations (37) and (38) can be written as:

(43) $$\Delta {\bar{V}}^{i+i_{1}}\approx \sum_{j=1}^{MKd}\kappa_{j}^{i_{1}}f_{j}e_{j}$$

(44) $$\Delta {\bar{U}}^{i+i_{1}}\approx \sum_{j=1}^{MKd}\kappa_{j}^{i_{1}}h_{j}g_{j}$$

According to Property 1, when *i*_1_ is large enough, it can be obtained that:

(45) $$\kappa_{1}^{i_{1}}\gg \kappa_{2}^{i_{1}}\gg ...\gg \kappa_{r}^{i_{1}},\kappa_{r+1}^{i_{1}}=\kappa_{r+2}^{i_{1}}=...=\kappa_{MKd}^{i_{1}}=0$$

Therefore, Equations (43) and (44) can be approximated by:

(46) $$\Delta {\bar{V}}^{i+i_{1}}\approx \kappa_{1}^{i_{1}}f_{1}e_{1}$$

(47) $$\Delta {\bar{U}}^{i+i_{1}}\approx \kappa_{1}^{i_{1}}h_{1}g_{1}$$

At the (*i* + *i*_1_ + 1)-th iteration, it can be obtained that:

(48) $$\Delta {\bar{V}}^{i+i_{1}+1}\approx \kappa_{1}^{i_{1}+1}f_{1}e_{1}\approx \kappa_{1}\Delta {\bar{V}}^{i+i_{1}}$$

(49) $$\Delta {\bar{U}}^{i+i_{1}+1}\approx \kappa_{1}^{i_{1}+1}h_{1}g_{1}\approx \kappa_{1}\Delta {\bar{U}}^{i+i_{1}}$$

From Equations (11), (12), (48) and (49), we have:

(50) $$\bar{\tilde{V}}={\bar{V}}^{i+i_{1}+1}-\Delta {\bar{V}}^{i+i_{1}+1}\approx {\bar{V}}^{i+i_{1}+1}+\frac{\kappa_{1}}{1-\kappa_{1}}(\Delta {\bar{V}}^{i+i_{1}+1}-\Delta {\bar{V}}^{i+i_{1}})$$

(51) $$\bar{\tilde{U}}\;={\bar{U}}^{i+i_{1}+1}-\Delta {\bar{U}}^{i+i_{1}+1}\;\approx {\bar{U}}^{i+i_{1}+1}+\frac{\kappa_{1}}{1-\kappa_{1}}(\Delta {\bar{U}}^{i+i_{1}+1}-\Delta {\bar{U}}^{i+i_{1}})$$

Define *t* = *κ*_1_/(1 − *κ*_1_) ∈ R and replace *i* + *i*_1_ + 1 with *i*, it can be obtained that:

(52) $$\begin{array}{c} \bar{\tilde{V}}\approx {\bar{V}}^{i}+t(\Delta {\bar{V}}^{i}-\Delta {\bar{V}}^{i-1})\\ ={\bar{V}}^{i}+t({\bar{V}}^{i}-{\bar{V}}^{i-1}) \end{array}$$

(53) $$\begin{array}{c} \bar{\tilde{U}}\approx {\bar{U}}^{i}+t(\Delta {\bar{U}}^{i}-\Delta {\bar{U}}^{i-1})\\ ={\bar{U}}^{i}+t({\bar{U}}^{i}-{\bar{U}}^{i-1}) \end{array}$$

Substituting Equations (10) into Equations (52) and (53), then Equations (35) and (36) can be obtained. Therefore the difference between the consecutive iteration results of AMIL algorithm, *i.e.*, $V_{k}^{i}-V_{k}^{i-1}$ and $U_{k}^{i}-U_{k}^{i-1}$ will approximately point to their convergence values ${\tilde{V}}_{k}$ and${\tilde{U}}_{k}$, when $V_{k}^{i}$ and $U_{k}^{i}$ are sufficiently close to ${\tilde{V}}_{k}$ and ${\tilde{U}}_{k}$, respectively.

Theorem 2 provides an enlightenment to reach the convergence point more rapidly. Instead of going along the circuitous route of VU-step and UV-step of AMIL algorithm, we can approach the destination more directly by searching along the new direction, *i.e.*, $V_{k}^{i}-V_{k}^{i-1}$ and $U_{k}^{i}-U_{k}^{i-1}$.This interesting discovery inspires us to propose the rapid convergent IA algorithm which will be shown in the next section.

## 4. Directional Quartic Optimal Algorithm

The traditional AMIL algorithm suffers from high complexity when there are plenty of users and antennas. The large number of iterations and long computational time required by the AMIL algorithm limit its application to practical IA systems where the CSI is always changing. Therefore, a high efficiency algorithm is needed to reduce the computational cost. In this section, we focus on a rapid convergent low-complexity IA approach, and propose the DQO algorithm. As shown in Section 3, the direction attained from the difference of the consecutive iteration results of the AMIL algorithm serves as a good searching direction, through which we can go to the convergence point almost directly. In addition, the optimal step size can be calculated by solving a quartic optimization problem. In this section, the details of the proposed algorithm are provided, and the associated computational complexity is analyzed.

### 4.1. The Procedure of DQO Algorithm

The procedure of DQO algorithm is summarized in Algorithm 2. The framework of DQO algorithm is based on the AMIL algorithm, and the LS procedure is added. In the initial phase of DQO algorithm, it operates just like the AMIL algorithm. The VU and UV steps are executed, and the interference leakage *L* is evaluated in each iteration. When *L* is smaller than the preset threshold *th*_1_, as shown in the 21-th line of Algorithm 2, the variable *flag* is set and the current number of iterations is recorded as *i*_b_, indicating that the current point is close enough to the convergence value and the LS procedure can be implemented. Notice that *flag* and *i*_b_ are updated only once and will remain unchanged afterward. After *flag* is set, the algorithm will execute the LS procedure every *interval* iterations, as shown in the 10-th line. The LS procedure cannot be carried out in every iteration for the reason that the approximations Equations (46) and (47) can be attained only when *i*_1_ is large enough.

**Algorithm 2** DQO Algorithm
Initialize $V_{k}^{0}$, with ${\left(V_{k}^{0}\right)}^{\text{H}}V_{k}^{0}=I$ (*k* = 1, 2,…, *K*).Initialize parameters: *th*_1_, *th*_2_ (*th*_2_ <* th*_1_), *I*_max_, and *interval*.Set *flag *= 0, *i *= 0, and *i*_b_ = 0.**Repeat***i *=* i *+ 1.(VU-step):Calculate $Q_{k}^{i}$ according to Equation (2). Update $U_{k}^{i}$ so that$U_{k(l)}^{i}={\text{eig}}_{l}[Q_{k}^{i}],l=1,2,..,d.$(UV-step):Calculate $\overset{\leftarrow }{Q_{k}^{i}}$ according to Equation (3). Update $V_{k}^{i}$ so that$V_{k(l)}^{i}={\text{eig}}_{l}[\overset{\leftarrow }{Q_{k}^{i}}],l=1,2,..,d.$
**If** *flag* = 1 and (*i *−* i*_b_) mod *interval *= 0, execute the LS procedure as:(a) Calculate the new iteration direction as:$\Delta U_{k}=U_{k}^{i}-U_{k}^{i-1}$
$\Delta V_{k}=V_{k}^{i}-V_{k}^{i-1}$
(b) Calculate the optimal step size t^*^.(c) Update $V_{k}^{i}$ and $U_{k}^{i}$ as:$U_{k}^{i}\leftarrow U_{k}^{i}\text{+}t^{*}\Delta U_{k}$$V_{k}^{i}\leftarrow V_{k}^{i}\text{+}t^{*}\Delta V_{k}$(d) Normalize each column of $V_{k}^{i}$ and $U_{k}^{i}$.**else**Calculate the interference leakage *L* according to Equation (5)**If** *th*_2_ <* L *<* th*_1_ and *flag *=0Set *flag *= 1 and *i*_b_ =* i*.**end if**.**end** **if**.**Until** *L* < *th*_2_ or *i* > *I*_max_.


Therefore, it has to take several UV and VU steps before $\kappa_{1}^{i_{1}}$ takes the dominant proposition so that Equations (35) and (36) hold. As a result, the parameter *interval* cannot be set to be one, and it will be further validated by simulation in Section 5.1. During each LS procedure, the new direction is calculated by subtracting the consecutive iteration results. The optimal step size t^*^ can be obtained by solving a quartic optimization problem, and the associated details will be provided in Section 4.2. The new precoders and decoders are updated as the 16-th and 17-th lines. To guarantee the unit norm of the precoders and decoders, we normalize each column of **U** and **V** in the 18-th line. The algorithm will stop when the interference leakage is smaller than the objective threshold *th*_2_ or the maximal iterations number *I*_max_ is reached.

### 4.2. Optimal Step Size Calculation

The step size in DQO algorithm is an essential parameter which has a significant impact on the efficiency. Aiming at minimizing the interference leakage along the line search direction, the optimal step size can be calculated analytically by solving a quartic optimization problem. As is shown in Equations (35) and (36), the step size *t* should be a real value. From Equations (2), (4) and (5), the total interference leakage in the case of **U***_k_* + *t*Δ**U***_k_*, **V***_k_* + *t*Δ**V***_k_* (*k* = 1, 2, …, *K*) can be formulated as:

(54) $$L(t)=\sum_{k=1}^{K}\sum_{l=1,l\neq k}^{K}\frac{P_{l}}{d}\text{Tr}[(U_{k}^{\text{H}}+t\Delta U_{k}^{\text{H}})H_{kl}(V_{l}+t\Delta V_{l})(V_{l}^{\text{H}}+t\Delta V_{l}^{\text{H}})H_{kl}^{\text{H}}(U_{k}+t\Delta U_{k})]$$

Without loss of generality, we assume *P_l_*/*d* = 1 and define:

(55) $$\alpha_{kl}=\Delta U_{k}^{\text{H}}H_{kl}V_{l}$$

(56) $$\beta_{kl}=\Delta U_{k}^{\text{H}}H_{kl}\Delta V_{l}$$

(57) $$\gamma_{kl}=U_{k}^{\text{H}}H_{kl}\Delta V_{l}$$

(58) $$\delta_{kl}=U_{k}^{\text{H}}H_{kl}V_{l}$$

Then Equation (54) can be formulated as a real quartic function of *t*:

(59) $$L(t)=a_{4}t^{4}+a_{3}t^{3}+a_{2}t^{2}+a_{1}t^{1}+a_{0}$$

where:

(60) $$a_{4}=\sum_{k=1}^{K}\sum_{l=1,l\neq k}^{K}\text{Tr}[\beta_{kl}\beta_{kl}^{\text{H}}]$$

(61) $$a_{3}=\sum_{k=1}^{K}\sum_{l=1,l\neq k}^{K}2\text{Re}\{\text{Tr}[\alpha_{kl}\beta_{kl}^{\text{H}}+\gamma_{kl}\beta_{kl}^{\text{H}}]\}$$

(62) $$a_{2}=\sum_{k=1}^{K}\sum_{l=1,l\neq k}^{K}\left\{\text{Tr}[\alpha_{kl}\alpha_{kl}^{\text{H}}+\gamma_{kl}\gamma_{kl}^{\text{H}}]+2\text{Re}\{\text{Tr}[\alpha_{kl}\gamma_{kl}^{\text{H}}+\beta_{kl}\delta_{kl}^{\text{H}}]\}\right\}$$

(63) $$a_{1}=\sum_{k=1}^{K}\sum_{l=1,l\neq k}^{K}2\text{Re}\{\text{Tr}[\delta_{kl}\alpha_{kl}^{\text{H}}+\delta_{kl}\gamma_{kl}^{\text{H}}]\}$$

(64) $$a_{0}=\sum_{k=1}^{K}\sum_{l=1,l\neq k}^{K}\text{Tr}[\delta_{kl}\delta_{kl}^{\text{H}}]$$

In order to obtain the global minimum value of Equation (59), the following lemma is firstly introduced:

**Lemma** **1.** For a real quartic function f(t) = a_4_t^4^ + a_3_t^3^ + a_2_t^2^ + a_1_t + a_0_, t ∈ R, with a_4_ > 0, there exists an optimal t^*^ ∈ R that satisfies f′(t^*^) = 0, so that f(t^*^) is the global minimal value of f(t).

**Proof** **of Lemma 1.** As *a*_4_ > 0 and $f^{\prime }(t)=4a_{4}t^{3}+3a_{3}t^{2}+2a_{2}t+a_{1}$, it can be obtained that $\underset{t\to +\infty }{\lim}f^{\prime }(t)=+\infty$ and$\underset{t\to -\infty }{\lim}f^{\prime }(t)=-\infty$. Therefore, there exist *a*, *b* ∈ R, so that *f*′(*t*) > 0, *t* ∈ [*b*, ∞) and *f′*(*t*) < 0, *t* ∈ (−∞ ,*a*]. Thus, *f*(*t*) increases monotonously in *t* ∈ [*b*, ∞) and has the minimal value *f*(*b*) at *t* = *b*. Similarly, *f*(*t*) decreases monotonously in *t* ∈ (−∞ ,*a*] and has the minimal value *f*(*a*) at *t* = *a*. When *t* ∈ [*a*, *b*], due to continuity, *f*(*t*) must have the minimal value *f*(*c*) at *t* = *c* where *c* ∈ [*a*, *b*]. Hence, min{*f*(*a*), *f*(*b*), *f*(*c*)} is the global minimum of *f*(*t*). In conclusion, there exists t^*^ ∈ R (or t^*^ ∈ {*a*, *b*, *c*}) so that *f*(t^*^) is the global minimal value. For *f*(*t*) ≥ *f*(t^*^), the left and right derivatives at t^*^ can be formulated as:

$$f_{-}^{\prime }(t^{*})=\underset{\Delta t\to 0^{-}}{\lim}\frac{f(t^{*}+\Delta t)-f(t^{*})}{\Delta t}\leq 0$$

$$f_{+}^{\prime }(t^{*})=\underset{\Delta t\to 0^{+}}{\lim}\frac{f(t^{*}+\Delta t)-f(t^{*})}{\Delta t}\geq 0$$

For $f^{\prime }(t^{*})=f_{-}^{\prime }(t^{*})=f_{+}^{\prime }(t^{*})$, we must have $f^{\prime }(t^{*})=f_{-}^{\prime }(t^{*})=f_{+}^{\prime }(t^{*})=0$. Therefore, the global optimal value t^*^ must be chosen from the solutions that satisfy *f*′(t^*^) = 0. When *f*′(t^*^) = 0 has only one real solution, it is the optimal t^*^. When *f*′(t^*^) = 0 has 2 or 3 real solutions, t^*^ is the one that has the smallest function value.

From Equation (60) we have *a*_4_ > 0. According to Lemma 1, it can be deduced that there exists t^*^ ∈ R with zero derivative, which makes *L*(t^*^) as the global minimal value of Equation (59). Let:

(65) $$dL/dt=4a_{4}t^{3}+3a_{3}t^{2}+2a_{2}t+a_{1}=0$$

The discriminant of Equation (65) is: $\Delta =\Delta_{1}^{2}+\Delta_{2}^{3}$, where Δ_1_ = (*bc*)/(6*a*^2^) − *b*^3^/(27*a*^3^) − *d*/(2*a*), Δ_2_ = *c*/(3*a*) − *b*^2^/(9*a*^2^), *a* = 4*a*_4_, *b* = 3*a*_3_, *c* = 2*a*_2_, and *d* = *a*_1_. And the 3 solutions of Equation (65) are given by:

(66) $$t_{\text{1}}\text{ = }x_{\text{1}}\text{+}x_{\text{2}}\text{+}x_{\text{3}}$$

(67) $$t_{2}=x_{1}+\frac{(-1+\sqrt{3}i)x_{2}}{2}+\frac{(-1-\sqrt{3}i)x_{3}}{2}$$

(68) $$t_{3}=x_{1}+\frac{(-1-\sqrt{3}i)x_{2}}{2}+\frac{(-1+\sqrt{3}i)x_{3}}{2}$$

where *x*_1_ = −*b*/(3*a*), $x_{2}=\sqrt[{3}]{\Delta_{1}+\sqrt{\Delta }}$, and $x_{3}=\sqrt[{3}]{\Delta_{1}-\sqrt{\Delta }}$. When Δ > 0 or Δ = Δ_1_ = Δ_2_ = 0, there is only one real root *t*_1_ which is the optimal t^*^; when Δ = 0 and Δ_1_ = Δ_2_ ≠ 0, there are 2 real roots; when Δ < 0, there are 3 real roots. If there are more than one real root, t^*^ is the one that has the smallest function value.

### 4.3. Computational Complexity Analysis

The computational complexity of the AMIL and DQO algorithms is analyzed according to the number of complex multiplications (NoCM). For the AMIL algorithm, the complexity comes from the covariance matrices calculation, eigenvalue decomposition, and interference leakage evaluation with the complexity of *K*(*K* − 1)(*MNd* + *Md*^2^ + *Nd*^2^), 9*K*(*M*^3^ + *N*^3^), and *K*(*K* − 1)(*d*^2^*M* + *d*^3^), respectively [17]. Therefore the NoCM per iteration of the AMIL algorithm is summarized as:

(69) $$C_{\text{AMIL}}=K(K-1)(2MNd+Nd^{2}+M^{2}d)+9K(N^{3}+M^{3})+K(K-1)(d^{2}M+d^{3})$$

For the DQO algorithm, the complexity of the VU-step, UV-step, and interference leakage calculations are the same as those of the AMIL algorithm, and the extra complexity comes from the LS procedure. The complexity of LS comes from the coefficients calculation of Equations (55)–(58) and Equations (60)–(63), solving Equation (65), and normalization. The complexity of each LS procedure is analyzed as follows:

(1) As shown in Equation (55), **α***_kl_* can be rewritten as $\alpha_{kl}=[{\left(U_{k}^{i}\right)}^{\text{H}}H_{kl}-{\left(U_{k}^{i-1}\right)}^{\text{H}}H_{kl}]V_{l}$. Since ${\left(U_{k}^{i}\right)}^{\text{H}}H_{kl}$ and ${\left(U_{k}^{i-1}\right)}^{\text{H}}H_{kl}$ have been calculated in ${\overset{\leftarrow }{Q}}_{k}$ as shown in Equation (3), the complexity of computing **α***_kl_* only comes from the multiplication of $\left[{\left(U_{k}^{i}\right)}^{\text{H}}H_{kl}-{\left(U_{k}^{i-1}\right)}^{\text{H}}H_{kl}\right]$ and $V_{l}$, with the NoCM of *d*^2^*M*. As *k* and *l* traverse from 1 to *K* with *l* ≠ *k*, the total complexity of calculating all **α***_kl_* is *K*(*K* − 1)*d*^2^*M*. Similarly, **β***_kl_*, **γ***_kl_*, and **δ***_kl_* have the same complexity as **α***_kl_*.

(2) The complexity of calculating *a*_1_–*a*_4_ mainly comes from the multiplications among **α***_kl_*, **β***_kl_*, **γ***_kl_*, and **δ***_kl_*. Notice that only the traces of the products are required, and we don’t have to compute all the elements of the products. Therefore, the complexity of calculating *a*_1_–*a*_4_ are 2*K*(*K* − 1)*d*^2^, 4*K*(*K* − 1)*d*^2^, 2*K*(*K* − 1)*d*^2^, and *K*(*K* − 1)*d*^2^, respectively. Notice that there is no need to calculate *a*_0_.

(3) The number of real solutions of Equation (65) depends on Δ_1_, Δ_2_ as well as Δ, and we consider the most complex case of 3 real solutions. The details of the complexity of solving the cubic equation are listed in Table 1. NoRM, NoRD, NoSRC, and NoCRC are employed to represent the number of real multiplications, real divisions, square root calculations, and cubic root calculations, respectively.

**Table 1:** Complexity of solving the cubic Equation (65).

Types	NoRM	NoRD	NoSRC	NoCRC
*a*, *b*, *c*, *d*	3	0	0	0
Δ_1_	9	3	0	0
Δ_2_	4	2	0	0
Δ	3	0	0	0
*x*_1_	1	1	0	0
*x*_2_	0	0	1	1
*x*_3_	0	0	1	1
*t*_1_	0	0	0	0
*t*_2_	8	0	0	0
*t*_3_	8	0	0	0
*L*(*t*)	21	0	0	0
Total	57	6	2	0

(4) $V_{k}^{i}$ and $U_{k}^{i}$ are normalized so that the norm of each column is one. The normalization of one column of $V_{k}^{i}$ takes *M* complex multiplications, one square root calculation and two real divisions. Therefore the normalization of all the precoding matrices takes *dMK* complex multiplications, *dK* square root calculations, and 2*dK* real divisions. Similarly, the normalization of all the decoding matrices takes *dNK* complex multiplications, *dK* square root calculations, and 2*dK* real divisions.

(5) As the complexity of one complex multiplication equals four real multiplications, the number of real multiplications will be replaced with the equivalent number of complex multiplications. And the complexity of one line search is summarized in Table 2.

Therefore the total number of equivalent complex multiplications of one line search is:

(70) $$C_{\text{LS}}=4K(K-1)d^{2}M+9K(K-1)d^{2}+dK(M+N)+14.25$$

And the number of real divisions, square root calculations and cubic root calculations in one LS are 4*dK* + 6, 2*dK* + 2, and 2, respectively. As LS is implemented every *interval* iteration, the average NoCM per iteration of DQO algorithm is:

(71) $$C_{\text{DQO}}=C_{\text{AMIL}}+C_{\text{LS}}/interval$$

**Table 2:** Complexity of one line search.

Types	NoRM	NoRD	NoSRC	NoCRC
α*_kl_*	*K*(*K* − 1)*d*^2^*M*	0	0	0
β*_kl_*	*K*(*K* − 1)*d*^2^*M*	0	0	0
γ*_kl_*	*K*(*K* − 1)*d*^2^*M*	0	0	0
δ*_kl_*	*K*(*K* − 1)*d*^2^*M*	0	0	0
*a*_1_	2*K*(*K* − 1)*d*^2^	0	0	0
*a*_2_	4*K*(*K* − 1)*d*^2^	0	0	0
*a*_3_	2*K*(*K* − 1)*d*^2^	0	0	0
*a*_4_	*K*(*K* − 1)*d*^2^	0	0	0
Solve (65)	14.25	6	2	2
Normalization	*dK*(*M* + *N*)	4*dK*	2*dK*	0

The complexity of the AMIL and DQO algorithms with the parameter *interval* = 20 (the mechanism for determining the parameter will be provided in Section 5.1) is compared in Table 3. From the comparison, it can be seen that the average NoCM of the DQO algorithm per iteration is only slightly higher than that of the AMIL algorithm. Although LS will bring extra complexity per iteration, it can reduce the number of iterations significantly and thus lower the overall computational complexity, which will be shown in the numerical results.

**Table 3:** Complexity of AMIL and DQO algorithms in (*M* × *M*, *d*)*^K^* with *interval* = 20.

(*M* × *N*, *d*)*^K^*	*C*_AMIL_	*C*_DQO_	*C*_DQO_/*C*_AMIL_
(6 × 6, 1)^11^	5.61 × 10^4^	5.63 × 10^4^	1.003
(6 × 6, 2)^5^	2.49 × 10^4^	2.50 × 10^4^	1.006
(7 × 7, 1)^13^	1.06 × 10^5^	1.06 × 10^5^	1.003
(7 × 7, 2)^6^	4.78 × 10^4^	4.80 × 10^4^	1.005
(8 × 8, 1)^15^	1.82 × 10^5^	1.83 × 10^5^	1.002
(8 × 8, 2)^7^	8.37 × 10^4^	8.40 × 10^4^	1.004
(9 × 9, 1)^17^	2.94 × 10^5^	2.95 × 10^5^	1.002
(9 × 9, 2)^8^	1.37 × 10^5^	1.37 × 10^5^	1.004
(10 × 10, 1)^19^	4.52 × 10^5^	4.53 × 10^5^	1.002
(10 × 10, 2)^9^	2.12 × 10^5^	2.12 × 10^5^	1.003

## 5. Numerical Results

In this section, the performance of the proposed DQO algorithm is evaluated and compared with the traditional AMIL algorithm by simulation. We will employ the interference leakage and sum rate as the performance metrics. The definition of interference leakage is shown in Equation (5), and that of sum rate can be found in reference [23]. Unless specially specified, 250 realizations of different channel coefficients with distribution CN(0, 1) and the corresponding initial precoding matrices are randomly generated to evaluate the average interference leakage and sum rate of the algorithms in each simulation.

### 5.1. Parameter Analysis

There are four parameters in the DQO algorithm: the interference leakage threshold for starting LS *th*_1_, the desired interference leakage *th*_2_, the interval for executing the LS *interval*, and the maximum iteration *I*_m_. The parameters *th*_2_ and *I*_m_ are set according to the practical requirements. The other two have impacts on the efficiency of DQO algorithm. It is difficult to provide the exact optimal values of the two parameters analytically. Therefore, simulations are employed to determine the parameters *th*_1_ and *interval* in this section.

**Figure 2:**
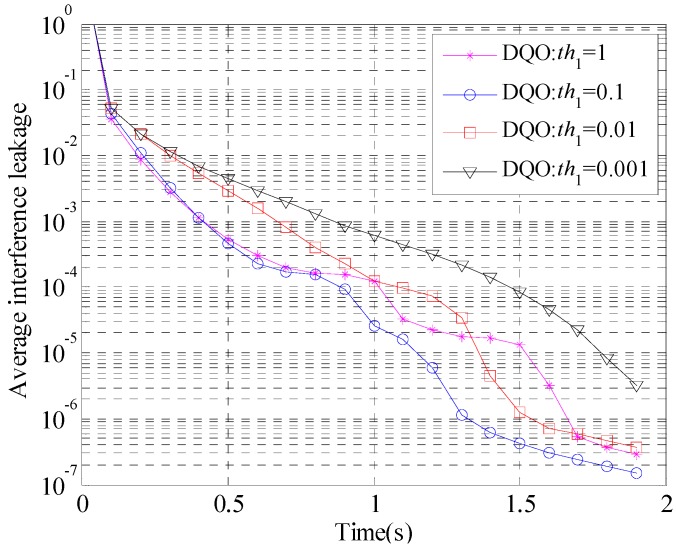
Convergence curves of the average interference leakage of DQO algorithm with *th*_1_ = 1, 0.1, 0.01, and 0.001 in (5 × 5, 2)^4^.

**Figure 3:**
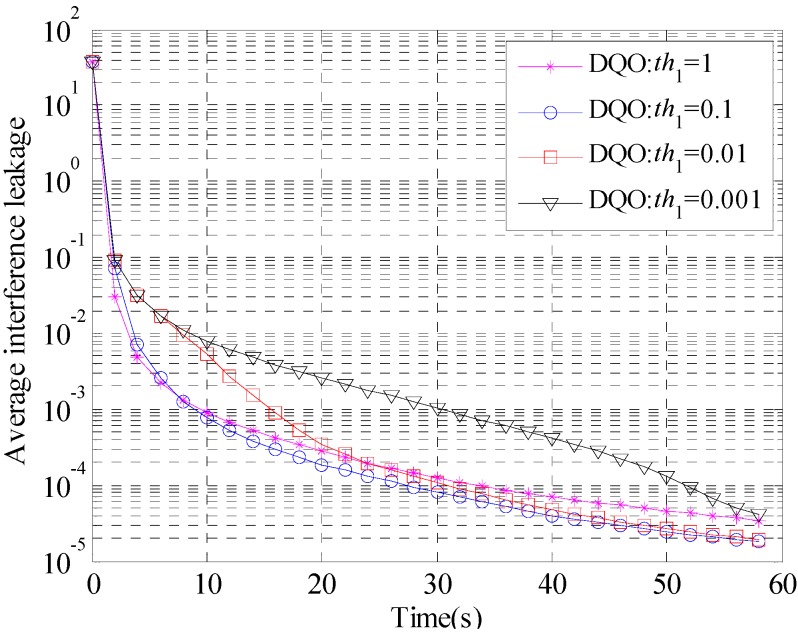
Convergence curves of the average interference leakage of DQO algorithm with *th*_1_ = 1, 0.1, 0.01, and 0.001 in (10 × 10, 1)^19^.

The parameter *th*_1_ decides the interference leakage threshold for starting line search. It is difficult to provide the exact optimal value of *th*_1_, and the performance of DQO algorithm is evaluated with different *th*_1_ for the (5 × 5, 2)^4^ and (10 × 10, 1)^19^ channels in Figure 2 and Figure 3, respectively. The parameter *interval* is preseted as 20, and *th*_1_ is chosen to be 1, 0.1, 0.01, and 0.001.

As is shown in Theorem 2, the difference between the two consecutive iteration results of AMIL algorithm will approximately point to the convergence solution when the current precoding and decoding matrices are sufficiently close to their convergence values. In DQO algorithm, the interference leakage is employed to be a measure of the distance between the current point and the convergence point, and line search is started when the interference leakage is smaller than a preseted threshold *th*_1_. If *th*_1_ is set to be too large, the generated direction does not point to the convergence value. If *th*_1_ is set to be too small, the line search procedure cannot accelerate the convergence rate in time, and the effectiveness of DQO algorithm is degraded.

As is shown in Figure 2 and Figure 3, *th*_1_ = 0.1 has better performance than the too large value 1 and the too small value 0.001, which accords with the analysis above. Therefore, the parameter *th*_1_ is set to be 0.1 in the following simulations.

The parameter *interval* controls the frequency of executing line search. The parameter cannot be set to be too small. This can be explained by the fact that Equations (46) and (47) can be attained only when *i*_1_ in Equation (45) is large enough. Therefore, it has to take several UV and VU steps before $\kappa_{1}^{i_{1}}$ in Equation (45) takes the dominant proposition so that Equations (35) and (36) hold.

**Figure 4:**
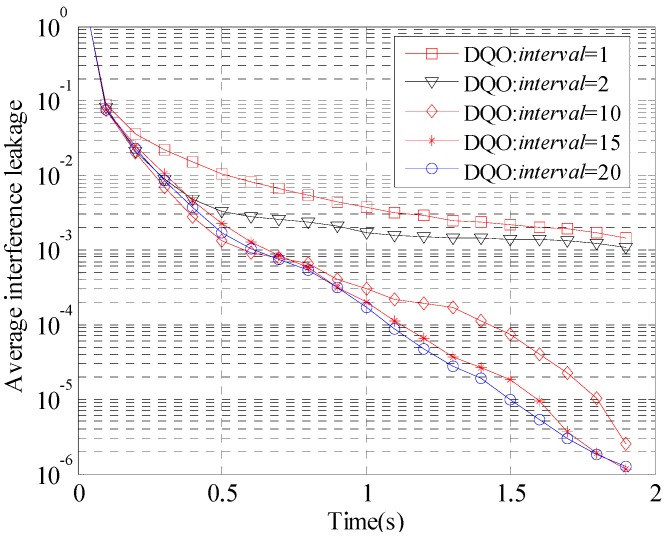
Convergence curves of the average interference leakage of DQO algorithm with *interval* = 1, 2, 10, 15, and 20 in (5 × 5, 2)^4^.

**Figure 5:**
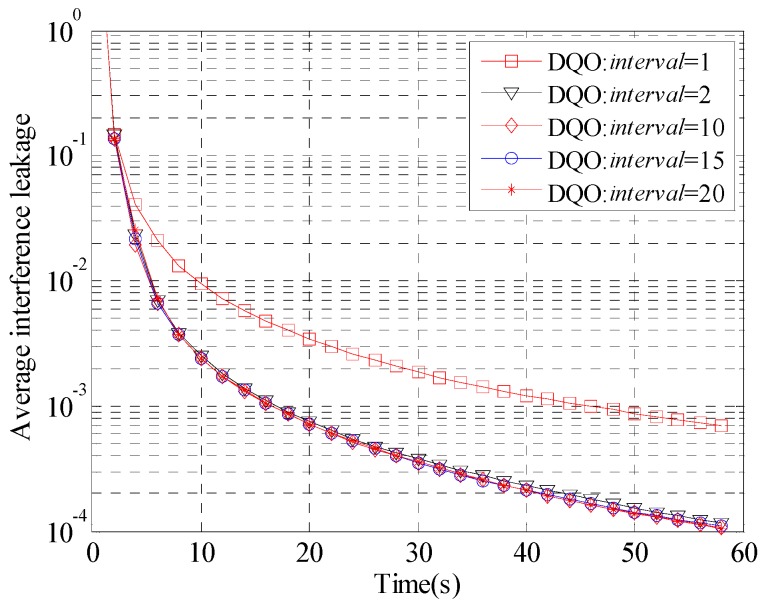
Convergence curves of the average interference leakage of DQO algorithm with *interval* = 1, 2, 10, 15, and 20 in (10 × 10, 1)^19^.

The performance of DQO algorithm with different *interval* for the (5 × 5, 2)^4^ and (10 × 10, 1)^19^ channels is evaluated in Figure 4 and Figure 5, respectively. The parameter *th*_1_ is preseted as 0.1, and *interval* is chosen to be 1, 2, 10, 15, and 20. The curves with *interval* = 1 converge slowly, which verifies the analysis above. On the other hand, when *interval* is large enough, the convergence curves are very close to each other. As *interval* = 20 has better performance than the others in Figure 4, *interval* is chosen to be 20 in the following simulations.

### 5.2. Comparison of Different Algorithms

**Figure 6:**
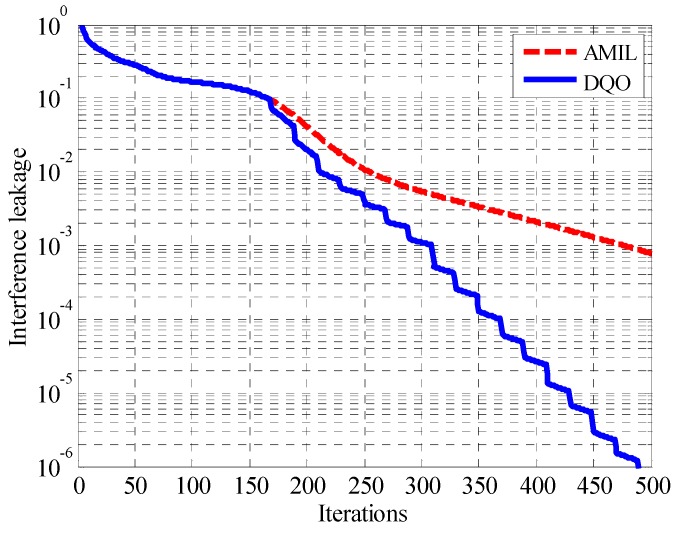
Convergence curves of the interference leakage for one realization of randomly generated channel coefficients in (5 × 5, 2)^4^.

In this subsection, the performance of the DQO and AMIL algorithms is compared and analyzed. The parameters for the DQO algorithm are set as *th*_1_ = 0.1 and *interval* = 20. The interference leakage with respective to iterations for one realization of randomly generated channel coefficients in the (5 × 5, 2)^4^ channel is shown in Figure 6, which provides us the overall effectiveness of DQO algorithm. As reflected in the figure, the interference leakage of the two algorithms remains the same before 168 iterations since they employ the same VU and UV steps. When the interference leakage is smaller than threshold *th*_1_, DQO algorithm begins to perform the LS procedure, and a step-like decrease in interference leakage can be observed. After 350 iterations, DQO algorithm reaches the interference leakage of 1.2 × 10^−4^, which is one order lower than that of AMIL algorithm. The difference of the interference leakage becomes larger as the iterations increase, and DQO algorithm can reach the level which is 3 orders lower than that of AMIL algorithm. Therefore, the DQO algorithm can reach the same level of interference leakage as the traditional AMIL method with much fewer iterations.

**Figure 7:**
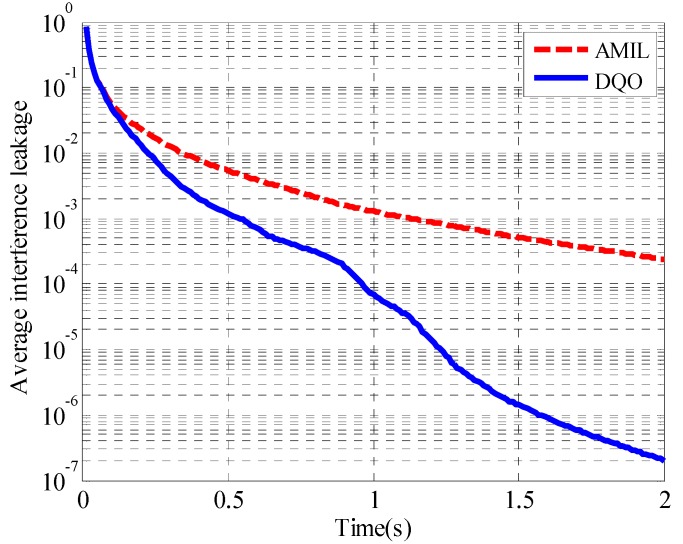
Convergence curves of the average interference leakage in (5 × 5, 2)^4^.

As iterations cannot represent the complexity of the two algorithms sufficiently, the average performance of the two algorithms is evaluated in terms of execution time in the following simulations. Average interference leakage as well as sum rate in (5 × 5, 2)^4^ and (10 × 10, 1)^19^ channels are considered. The convergence of average interference leakage for (5 × 5, 2)^4^ channel is illustrated in Figure 7. 

**Figure 8:**
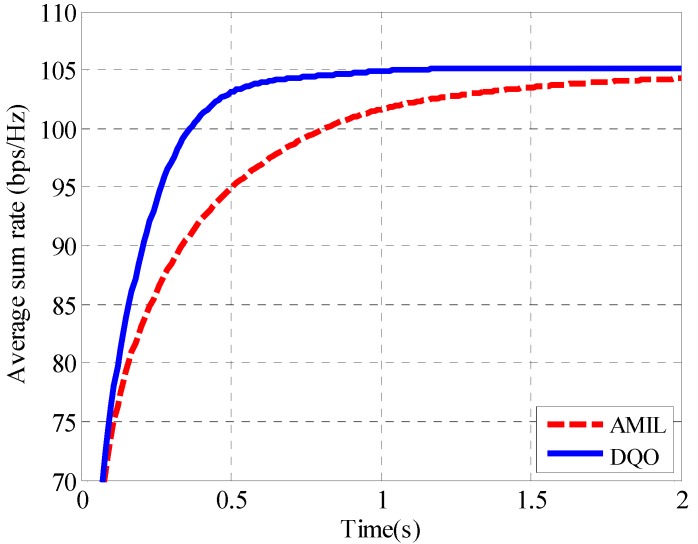
Convergence curves of the average sum rate in (5 × 5, 2)^4^.

From the results, it can be seen that the DQO algorithm can suppress the interference leakage to 6.8 × 10^−5^ while the AMIL algorithm can only reach the level of 1.2 × 10^−3^ at 1 s. The difference of the average interference leakage between them increases with time, and the DQO algorithm outperforms the AMIL algorithm with three orders lower at 2 s. The convergence of the average sum rate with SNR = 40 dB for (5 × 5, 2)^4^ channel is depicted in Figure 8. 

**Figure 9:**
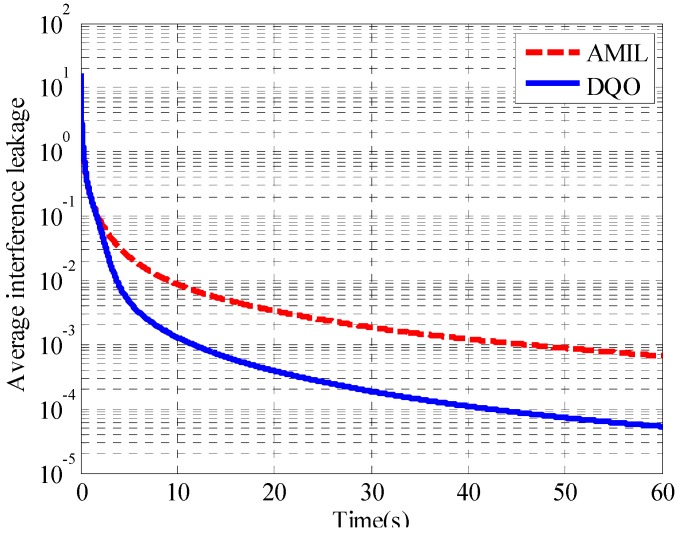
Convergence curves of the average interference leakage in (10 × 10, 1)^19^.

**Figure 10:**
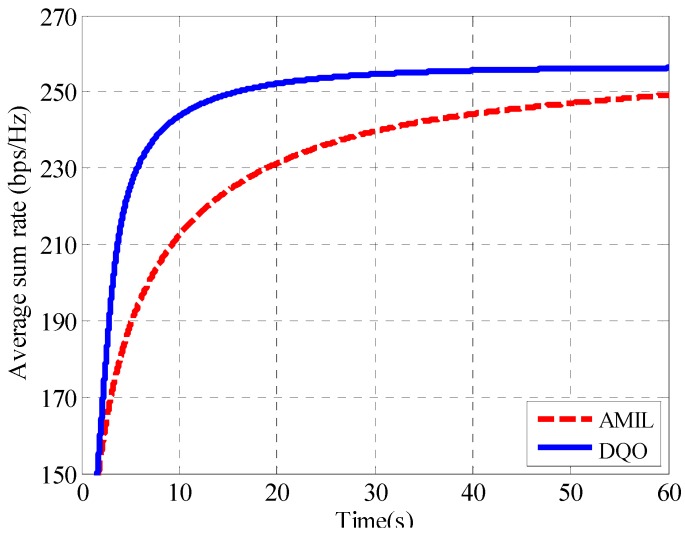
Convergence curves of the average sum rate in (10 × 10, 1)^19^.

As reflected in the figure, the average sum rate of the DQO algorithm increases more rapidly than that of the AMIL algorithm during the first 0.5 s and the DQO algorithm converges approximately at 0.61 s with the average sum rate of 104 bps/Hz while the AMIL algorithm takes about 1.78 s to reach the same level. Like the (5 × 5, 2)^4^ case, the convergence curves of average interference leakage as well as sum rate for (10 × 10, 1)^19^ channel are plotted in Figure 9 and Figure 10, respectively. As the number of users and antennas has become larger, it requires more iterations as well as computational time to suppress the interference leakage and to increase the sum rate. In Figure 9, the DQO algorithm can suppress the interference leakage more rapidly than the AMIL algorithm and achieves the interference leakage of 5.6 × 10^−5^ at 60 s, which is one order lower than that of the AMIL algorithm. In particular, the AMIL algorithm seems to converge at approximately 60 s while the DQO algorithm has the tendency to reach an even lower level of interference leakage afterward. In Figure 10, the average sum rate of the DQO algorithm converges much more rapidly than that of the AMIL algorithm during the first 10 s and the DQO algorithm achieves a higher average sum rate than the AMIL algorithm. To further compare their efficiency, average iterations N_AMIL_ and N_DQO_ as well as executing time *T*_AMIL_ and *T*_DQO_ with *th*_2_ = 10^−4^ are listed in the cases of different numbers of users and antennas in Table 4. Yetis *et al.* [32] have provided the feasible condition for IA in the (*M* × *N*, *d*)*^K^* channel as *d* ≤ (*M* + *N*)/(*K* + 1), therefore we mainly provide the results of the most complex situation when *d* = (*M* + *N*)/(*K* + 1). 

The convergence rate and complexity of the algorithms can be compared by the iterations and computational time which are required to reach a certain level of interference leakage. As is shown in Table 4, the iterations and executing time of the DQO algorithm are only 22%–33% and 23%–33% of those of the AMIL algorithm, respectively. Therefore, simulations above confirm that DQO algorithm can converge faster than the AMIL algorithm and save nearly 2/3 of the computational complexity.

**Table 4:** Comparison between AMIL and DQO algorithms in (*M* × *N*, *d*)*^K^.*

(*M* × *N*, *d*)^K^	N_AMIL_	N_DQO_	N_DQO_/N_AMIL_	*T*_AMIL_(s)	*T*_DQO_(s)	*T*_AMIL_/*T*_DQO_
(6 × 6, 1)^11^	3.38 × 10^3^	7.76 × 10^2^	0.22	17.12	3.89	0.23
(6 × 6, 2)^5^	2.63 × 10^3^	8.13 × 10^2^	0.31	4.09	1.27	0.31
(7 × 7, 1)^13^	4.93 × 10^3^	1.27 × 10^3^	0.25	35.75	9.03	0.25
(7 × 7, 2)^6^	4.60 × 10^3^	1.47 × 10^3^	0.32	9.94	3.18	0.32
(8 × 8, 1)^15^	7.23 × 10^3^	1.74 × 10^3^	0.24	64.26	15.35	0.24
(8 × 8, 2)^7^	6.16 × 10^3^	2.06 × 10^3^	0.33	17.75	5.91	0.33
(9 × 9, 1)^17^	8.35 × 10^3^	2.04 × 10^3^	0.24	99.33	24.16	0.24
(9 × 9, 2)^8^	8.12 × 10^3^	2.24 × 10^3^	0.27	30.61	8.41	0.27
(10 × 10, 1)^19^	9.44 × 10^3^	2.52 × 10^3^	0.27	140.12	36.71	0.27
(10 × 10, 2)^9^	8.94 × 10^3^	2.35 × 10^3^	0.26	42.49	11.10	0.26

## 6. Conclusions and Future Work

In this paper, the properties of the traditional AMIL algorithm for IA have been studied. It has been found that if the AMIL algorithm can converge to a point where the interference leakage is very small, there exist fixed linear transformations **T**_V_ and **T**_U_ that exert on the deviations when the current point is sufficiently close to the convergence value. Particularly, successive exertions of the transformations on the deviations will lead to an interesting property that the difference of the consecutive iterations of AMIL algorithm will approximately point to the convergence value. Based on this discovery, a rapid convergent low-complexity minimization interference leakage algorithm, namely the DQO algorithm, has been proposed to speed up the convergence rate by employing optimal line search procedure. The direction for LS is generated by subtracting the consecutive iteration results of the AMIL algorithm, and the optimal step size can be determined analytically by optimizing a quartic function. Complexity analysis of the DQO and AMIL algorithms has been provided, and simulation results have shown that the DQO algorithm can reduce the number of iterations and execution time significantly under the same interference leakage and sum rate conditions. In the future, we will focus on extending the proposed algorithm to many more scenarios, such as systems with imperfect CSI [33,34], cooperative communications [35], and so on.
